# Dietary Intake and Mental Health among Saudi Adults during COVID-19 Lockdown

**DOI:** 10.3390/ijerph18041653

**Published:** 2021-02-09

**Authors:** Hanan Alfawaz, Sobhy M. Yakout, Kaiser Wani, Ghadah A. Aljumah, Mohammed G. A. Ansari, Malak N. K. Khattak, Syed D. Hussain, Nasser M. Al-Daghri

**Affiliations:** 1 Department of Food Science & Nutrition, College of Food Science & Agriculture, King Saud University, Riyadh 11495, Saudi Arabia; halfawaz@ksu.edu.sa; 2 Chair for Biomarkers of Chronic Diseases, Biochemistry Department, King Saud University, Riyadh 11451, Saudi Arabia; sobhy.yakout@gmail.com (S.M.Y.); wani.kaiser@gmail.com (K.W.); ansari.bio.1@gmail.com (M.G.A.A.); malaknawaz@yahoo.com (M.N.K.K.); danishhussain141@gmail.com (S.D.H.); 3 King Abdulaziz Medical City, Riyadh 14611, Saudi Arabia; Aljumahgh@ngha.med.sa

**Keywords:** anxiety, COVID-19, depression, pandemic, Saudi Arabia

## Abstract

The study aimed to explore the influence of the COVID-19 lockdown on the mental status and dietary intake of residents in Saudi Arabia. In this cross-sectional study, an online survey was conducted from 11 May to 6 June 2020 corresponding to almost two weeks during and after Ramadan (23 April–23 May 2020). The Patient Health Questionnaire was used to assess anxiety, depression, and insomnia. Logistic regression analysis was used to identify predictors of anxiety, depression, and insomnia. The prevalence of anxiety, depression, and insomnia among the participants was 25.4%, 27.7%, and 19.6%, respectively. Participants aged ≥50 years with high income (≥8000 SAR) were at a lower risk of developing depression, whereas participants of the same age group with income 5000–7000 SAR were at high risk of developing anxiety. Students and master-educated participants suffer from median elevated depression and are required to take more multivitamins and vitamin D than others. Anxiety and depression were more common among married participants with low income. There is a wide range of Saudi residents who are at a higher risk of mental illness during the COVID-19 pandemic. Policymakers and mental healthcare providers are advised to provide continuous monitoring of the psychological consequences during this pandemic and provide mental support.

## 1. Introduction

A novel coronavirus was first identified from a cluster of Chinese patients diagnosed with unknown cases of pneumonia in December 2019 [1]. Tentatively known as 2019-nCoV, the virus is now named SARS-CoV2, and the term Covid-19 denotes the disease caused by SARS-CoV2 [2]. According to the World Health Organization (WHO), the most common symptoms of Covid-19 include fever (85% of cases), and 45% of cases develop dyspnea, dry cough, sore throat, nasal congestion, and lung infiltrates [3]. Approximately 10–15% of cases progress to severe disease, and about 5% become critically ill while the majority experiences mild to moderate symptoms and recovers within two to six weeks. However, symptoms may recur after initial recovery, even for people suffering from mild disease. The number of confirmed novel coronavirus (SARS-CoV2) deaths globally has surpassed two million [4], with worst outcomes coming from individuals with pre-existing conditions, including Saudi Arabia, where most hospitalized Covid-19 patients have diabetes [5].

Covid-19 research is a rapidly evolving field at a global level, and currently, the single most effective strategy is social distancing, decreasing the rate of infection by as much as 92% [2]. This practice has been in place in most countries affected, including other mitigation strategies, such as area lockdowns, curfews, and suspension of travel.

In Saudi Arabia, the government announced a series of extreme measures to control the spread of the virus after its first confirmed case by banning all transport in and out of the Qatif Governorate on 8 March and 6 April 2020, to announce a 24-h curfew implemented in the major cities with movement restricted to only essential travel between 6 a.m. and 3 p.m. [6]. The extremely proactive measures taken to prevent the spread of the virus have contributed to public fear, anxiety, and/or depression, which are usually neglected during crisis and pandemic management [7,8,9,10,11].

Psychological stress can be external and related to the environment [12], but may also be caused by internal perceptions that cause an individual to experience anxiety or other negative emotions surrounding a situation, such as pressure, discomfort, etc., which they then deem stressful. The rapid spread and consequential health effects of COVID-19, the coverage that this disease gets in print and social media, and the associated frightening statistics are likely to have heightened anxiety with adverse impact on mental health [13]. Such large-scale disasters, be it natural or man-made, have always had a negative impact on an individual’s psychological well-being [14]. Because of the novel nature of COVID-19, people equip themselves with new information from multiple sources, fueling more fear and anxiety [15]. Studies on the psychological effects, especially among front liners [16,17], and those who self-quarantined due to various reasons [18], may help identify practices that benefit the general public [19].

Most COVID-19 research focused on outcomes [5,20], and data on mental health are lacking [8,21]. COVID-19 was recently found to be associated with neurological damage [22]. Psychiatric symptoms, such as depression, can impair cognitive functioning [23,24] and work performance [25].

Studies have shown that a healthy diet and physical activity can translate to better mental health [26,27], and observational studies have reported that COVID-19 lockdown influenced dietary profiles [28,29]. These nutritional adaptations may boost the immune system, which can influence the host’s response to infection [30]. Micronutrients, such as vitamin D, and mineral deficiencies have long been a major healthcare problem in the Middle East [31,32]. Vitamin D has been shown to have antiviral and anti-inflammatory properties that are beneficial overall and more so in optimal pulmonary function [33,34]. It has also been shown that supplementation with vitamin D is a good preventive strategy in recurring respiratory tract infections [3] and also COVID-19 infections [20]. Furthermore, there is mounting evidence to suggest that balanced zinc, selenium, and vitamin C levels prevent viral respiratory infections [35,36,37,38,39,40]. Zinc is a dietary trace mineral that boosts immune cells [41]. Zinc deficiency leads to an extensive loss of immunity and increased risk of infectious diseases, and coronavirus is no exception [42].

The present study was thus aimed to investigate the prevalence of self-reported mental health conditions and their associations with micronutrient dietary intake and other sociodemographic factors among Saudi residents during the COVID-19 lockdown.

## 2. Materials and Methods

### 2.1. Study Design and Participants

Details of methodology have been published previously [43]. This cross-sectional online survey was designed to study the lifestyle changes and mental wellness of Saudi adults, Riyadh, Saudi Arabia, during the COVID-19 lockdown. This survey was conducted from 11 May to 6 June 2020. All adult Saudi citizens and residents (non-Saudis) 18 years old and above with access to the internet were deemed eligible to voluntarily participate in the survey. Participation was verified through email and we ensured that each participant completed the survey only once. Registered adults who completed the surveys were included in the data analysis, while those who did not complete the survey were excluded. The study included 958 adult men (450) and women (508) from Riyadh, Saudi Arabia. Height and weight were self-declared and were not independently measured. Body mass index (BMI) was calculated (kg/m^2^).

The study design and protocol were approved by the Ethics Committee for Scientific Research and Post Graduate Studies at the College of Science, King Saud University, Saudi Arabia (reference# KSU-HE-20-246).

### 2.2. Questionnaire

The questionnaire included a cover letter in Arabic and English. Experts in the related field reviewed the questionnaire and several revisions were made to strengthen the reliability and enhance the scientific value of the data to be collected. A pilot study (N = 75 participants) was performed to confirm the reliability and validity of the questionnaire and obtained Cronbach’s α, which was noted to be excellent (84%). After completion, the questionnaire was transferred to an online link for distribution to different social media outlets throughout Saudi Arabia.

The questionnaire consisted of three sections:

(1) Sociodemographic characteristics, including age, sex, marital status, family income, educational qualification, employment status, etc.
(2) Supplements used during the quarantine, including dietary supplements containing vitamin D, selenium, zinc, and vitamin C. Time exposure to sun and sleeping times were also included.
(3) Mental wellness of the participants (e.g., if the participants experienced anxiety, depression, and/or insomnia during the lockdown). In this section, the operational definition of these mental health conditions was given before the questions. “Depression” was defined as the condition in which a person experienced low mood and/or loss of interest in most activities for two weeks or longer with symptoms like tiredness, poor concentration, etc. “Anxiety” was defined as the condition in which a person experienced persistent and excessive worry with symptoms ranging from headaches, fast heartbeat, shortness of breath, etc. “Insomnia” was defined as a condition where a notable change in sleep patterns, difficulty in falling or staying asleep was observed. The options were scaled as “constant”, “sometimes”, and “never”.

### 2.3. Sample Size Calculation

Sample size calculation was done using Raosoft online to specify the number of respondents needed with an error margin to meet the desired confidence level. In order to obtain a confidence level of 95% and a 3.15% margin of error, a minimum sample size of 958 would enable us to achieve the study objectives.

### 2.4. Data Analysis

Analysis was done using SPSS version 16.5 (IBM, Chicago, IL, USA). Continuous variables were presented as mean ± standard deviation while categorical variables were presented as frequencies (N) and percentages (%). Uncorrelated errors and consistency in coding were confirmed prior to reliability analysis. Chi-Square test and independent t-test were performed to determine differences between categorical and continuous variables of interest on a gender basis. Multinomial logistic regression analysis was performed for dependent parameters anxiety, depression and insomnia. *p*-value was considered significant at *p* < 0.05 and 0.01 level.

## 3. Results

### 3.1. Sociodemographic Characteristics of Participants

Table 1 details the baseline characteristics of the participants. A total of 958 individuals participated in the study with the age of 36.7 ± 13.8 years and BMI of 26.6 ± 5.6 kg/m^2^ from Riyadh, Saudi Arabia. 47% (450) of the study participants were males, and 53% (508) were females. A large proportion of participants were educated either up to graduate or higher level (880, 92%). A fair representation from low (<5000 SR; 308, 32.2%), moderate (5000–7000 SR; 267, 27.9%), and high-income groups (>16,000 SR; 281, 29.3%) could be seen in the study participants, and there was no statistical difference between genders. There was a significant difference in the proportion of married participants between males (281, 29.3%) and females (255, 50.2%).

The majority of participants were Saudi (*n* = 779, 81.3%), males (*n* = 375, 83.3%), aged between 15 and 25 years (*n* = 267, 9.27%), married (*n* = 532, 5.55%), employed (*n* = 551, 5.57%), and having a bachelor degree (*n* = 541, 5.56%) with family monthly income >16,000 (*n* = 281, 29.3%). When participants were asked whether at first true sign of COVID-19 symptoms they isolated themselves, 88.2% (845) responded “Yes”, whereas, 84.2% (807) were still covering their mouth when coughing.

### 3.2. Prevalence of Mental Health Problems

Table 2 details the prevalence of micronutrient intake, depression, anxiety, and insomnia among the participants. The prevalence of anxiety, depression and insomnia among the participants was 25.4% (*n* = 243), 27.7% (*n* = 265), and 19.6% (*n* = 188), respectively. The proportions of subjects that used multivitamins, vitamin D, vitamin C, zinc, and selenium were 23.6% (*n* = 226), 20.1% (*n* = 193), 11.9% (*n* = 114), 0.9% (*n* = 9), and 0.3% (*n* = 3), respectively.

As seen in Table 3, participants aged above 36 years with high income (8000 SR and above) tend to have lower depression median scores compared to others. Student participants and those with a master education level suffer from high depression median and are forced to take more multivitamins and vitamin D more than others.

Participants with age 26–35 years with an income of 5000–7000 SR tend to have a higher anxiety median compared to others. They tend to take more vitamin D supplementation and sleep less. However, participants aged above 45 years tend to have less anxiety. Participants with age above 45 years with income 5000–7000 SR tend to have a higher anxiety median compared to others. They tend to take more multivitamins, vitamin D, and vitamin C supplementation and sleep less.

Logistic regression analysis identified the following groups to be at a higher risk of depression: (a) Education and employment status student (OR 2.07 (95% CI 1.07–4.02), *p* < 0.05) and OR 2.05 (95% CI, 1.48–2.84), *p* < 0.01), respectively), (b) multivitamin intake (OR 1.45 (95% CI, 1.04–2.02, *p* < 0.05)), and (c) vitamin D (OR 1.53 (95%CI, 1.08–2.16), *p* < 0.05) for depression. On the other hand, the following groups were at a lower risk of depression: (a) Age above 36 years and (b) individuals with high income (8000 SR and above) and sleeping hour increase. Moreover, logistic regression analysis showed that the following groups were at a higher risk of anxiety: (a) Unemployed individuals with low income less than 7000 SR, and (b) vitamin D 1.71 (1.22–2.39, *p* < 0.01). On the other hand, individuals with age above 45 years were at a lower risk of both anxiety and insomnia. Furthermore, logistic regression analysis in Table 3 shows that intake of multivitamins, vitamins C and D was linked to a higher risk of insomnia (*p* < 0.05) and no change in sleeping hours was a protective element for depression, anxiety, and insomnia, respectively (*p* < 0.01).

**Figure 1:**
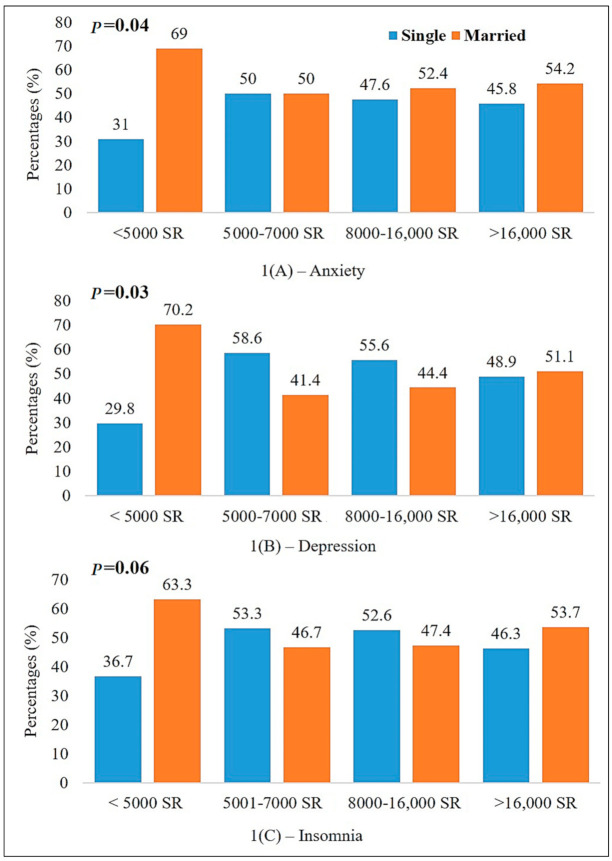
Prevalence of different mental health disorders according to income and marital status.

Lastly, Figure 1 shows the prevalence of mental disorders according to income and marital status and showed a significantly higher prevalence of anxiety (1A) and depression (1B) and borderline significance in insomnia (1C) among married participants whose income was below 5000 SR (*p*-values 0.04, 0.03, and 0.06, respectively).

## 4. Discussion

This cross-sectional survey observed a high prevalence of self-reported anxiety, depression, and insomnia among Saudi adults during the COVID-19 lockdown, experiencing these either constantly or occasionally. Overall, the findings in this study demonstrated that more than 25.4% of respondents had depression, whereas, the prevalence of anxiety and insomnia retches up to 7.27% and 19.6%, respectively.

During stressful conditions, such as the case with the COVID-19 pandemic, fear and anxiety about the disease can be overwhelming, which may cause depression and anxiety among adults and even children [44]. The sudden shutdown of services and lockdown of people are predisposing to such conditions, particularly when dealing with the unpredictable status of the outbreak. The fear of getting the disease and losing loved ones [45] is another predisposing factor that may result in such a condition. The prevalence rate of moderate-to-severe depression symptoms in this study seems to be considerably higher than that of those reported by the Chinese study that included 1210 respondents during the COVID-19 outbreak (16.5%), whereas comparable rates for anxiety were noted (28.8%) [5]. Moreover, it was observed that results vary according to the sample size and the used assessment tool. Where in another nationwide study among Chinese people during the pandemic including 52,730 participants revealed a psychological distress prevalence rate of 35% among all respondents. Distress symptoms according to the employed assessment tool included depression, anxiety, and insomnia [46].

Depression and anxiety symptoms were more likely to occur in men than in women. Such results agree with other studies that investigated depression and anxiety among the Saudi Arabian population. The results of a study conducted by Al-Khathami and co-workers (2002) reported that the prevalence of minor mental illness was significantly higher in women (22.2%) than men (13.7%) (*p* = 0.0073) [47]. Further, the prevalence rate was high in the younger age group, which agrees with our study, as a higher score of depression was associated with individuals younger than 29 years. Further, the study of Wang et al. (2020) revealed that the female gender was significantly associated with a greater psychological impact of the COVID-19 outbreak and had higher levels of stress, anxiety, and depression (*p* < 0.05) [1]. Several factors can contribute to women’s higher depression and anxiety prevalence rates, including biological sex differences, culture, diet, female hormonal fluctuations, and education [48].

Sociodemographic variables associated with mental health status were assessed using logistic regression analysis, which showed that the public’s levels of depression, anxiety, and insomnia-related symptoms increase among students and unemployed or those with low income. The results were in agreement with previous research, which showed that students were more likely to have depression and anxiety during the pandemic [6]. The onset of the pandemic was in the middle of the academic year, which contributed to students’ fear of losing the year or the occurrence of delays in their studies, besides the lack of confidence in remote learning. The closure and social distancing are anticipated to sustain for longer periods, and this apparently will have a direct effect on low income and unemployed individuals [49], and, therefore, this might put such categories under a higher level of stress that could lead to anxiety and/or depression symptoms.

Moreover, our results showed that people with depression, anxiety, and insomnia take significantly more vitamin D (Table 3 and Figure 1). Previous interventions have shown the impact of vitamin D deficiency on depression [50,51], however, limited studies have been carried out to illustrate the association of anxiety disorders with serum levels of vitamin D [52]. Penckofer et al. reported that six-month intervention with 50,000 IU vitamin D significantly decreased depression and anxiety in people with type 2 diabetes mellitus (T2DM) who had depression [53]. Supplementation with vitamin D reduced anxiety levels in premenstrual syndrome in adolescents who had severe vitamin D deficiency [54].

This study demonstrated several strengths. First, it addressed the prevalence of depression, anxiety, and insomnia within the initial phase of the pandemic and hence will provide valuable information to policymakers that will enable them to make informed decisions and introduce psychological interventions that can minimize psychological untoward effects and mental health status among Saudi Arabian people. Second, it involved a good sample size that was not limited to specific geographical areas in Saudi Arabia.

However, this study has several limitations. First, the study was based on a web-based survey method; therefore, some vulnerable individuals who have no access to the internet and are unfamiliar with online questionnaires were missed. Second, due to the sudden occurrence of the outbreak, an individuals’ depression, anxiety and insomnia prevalence before the outbreak could not be pursued. Third, the survey was administered at a single period and hence the stability of the responses is unknown. Lastly, the nature of the study being a cross-sectional and self-reported survey limits its findings to, at best, suggestive.

## 5. Conclusions

In conclusion, there is a high prevalence of self-reported anxiety, depression, and insomnia among Saudi adults during COVID-19 lockdown. During the initial phase of the COVID-19 pandemic in Saudi Arabia, more than 27% of the respondents had depression, about 25% reported anxiety, and more than 19% had insomnia. Individuals aged 50 years and above with high income were at a lower risk of developing depression, while those with lower income were at high risk of developing anxiety. Students and individuals with a master’s degree suffered from depression, individuals with lower income were at risk of developing anxiety. Anxiety and depression were also more common among married participants with low income. It was also noted that the majority of the respondents were not using any vitamins or other mineral supplements during the pandemic. Our findings may enable policymakers to introduce several measures and psychological interventions that can enhance mental health during the COVID-19 pandemic.

## Figures and Tables

**Table 1:** General information on the survey subjects.

Parameters	N (%)	Males	Females	*p*-Value
N	958	450	508	
Age (years)	36.7 ±13.8	36.6 ± 13.9	36.8 ± 13.8	0.850
Weight (kg)	73.6 ± 17.2	72.8 ± 16.2	74.3 ± 18.1	0.171
BMI (kg/m^2^)	26.6 ± 5.6	26.4 ± 5.3	26.8 ± 5.9	0.287
**Marital Status**				
Single	400 (41.8)	173 (38.4)	227 (44.7)	<0.001
Married	532 (55.5)	277 (61.6)	255 (50.2)	
Divorce	20 (2.1)	---	20 (3.9)	
Widow	6 (0.6)	---	6 (1.2)	
**Nationality**				
Saudi	779 (81.3)	375 (83.3)	404 (79.5)	0.080
Non-Saudi	179 (18.7)	75 (16.7)	104 (20.5)	
**Education**				
High School	78 (8.1)	29 (6.4)	49 (9.6)	0.262
Bachelor	541 (56.5)	261 (58.0)	280 (55.1)	
Master	173 (18.1)	78 (17.3)	95 (18.7)	
PhD	166 (17.3)	82 (18.2)	84 (16.5)	
**Family Monthly Income**				
Low (<5000)	308 (32.2)	153 (34.0)	155 (30.5)	0.452
Average (5000–7000)	102 (10.6)	42 (9.3)	60 (11.8)	
Moderate (8000–16,000)	267 (27.9)	121 (26.9)	146 (28.7)	
High (>16,000)	281 (29.3)	134 (29.8)	147 (28.9)	
**Employment Status**				
Employed	551 (57.5)	247 (54.9)	304 (59.8)	0.382
Unemployed	119 (12.4)	63 (14.0)	56 (11.0)	
Student	254 (26.5)	123 (27.3)	131 (25.8)	
Farmer	34 (3.5)	17 (3.8)	17 (3.3)	
**Age Group**				
15–25 year	267 (27.9)	129 (28.7)	138 (27.2)	0.810
26–35 year	248 (25.9)	110 (24.4)	138 (27.2)	
36–45 year	194 (20.3)	93 (20.7)	101 (19.9)	
>45 year	249 (26.0)	118 (26.2)	131 (25.8)	
**Isolate myself at the first genuine sign of COVID 19 symptoms**				
Yes	845 (88.2)	395 (87.8)	450 (88.6)	0.777
No	30 (3.1)	16 (3.6)	14 (2.8)	
Don’t Know	83 (8.7)	39 (8.7)	44 (8.7)	
**Always cover mouth when coughing since COVID 19 outbreak**				
Yes	807 (84.2)	380 (84.4)	427 (84.1)	0.984
No	104 (10.9)	48 (10.7)	56 (11.0)	
Don’t Know	47 (4.9)	22 (4.9)	25 (4.9)	

Note: Data presented N (%). *p*-value < 0.01, 0.05 was significant for chi-square and fisher exact test.

**Table 2:** Prevalence of micronutrient dietary supplements, depression, anxiety, and insomnia among the participants.

Parameters	Frequency
Suffered from anxiety during the quarantine	265 (27.7)
Suffered from depression during the quarantine	243 (25.4)
Suffered from insomnia	188 (19.6)
The supplement used during the quarantine	
Multivitamin	226 (23.6)
Vitamin D	193 (20.1)
Vitamin C	114 (11.9)
Zinc	9 (0.9)
Selenium	3 (0.3)

Note: Data presented N (%).

**Table 3:** Predictors for anxiety, depression, and insomnia.

Parameters	Anxiety	Depression	Insomnia
	OR (95%CI)	OR (95%CI)	OR (95%CI)
**Demographics**			
**Nationality**			
Saudi	1	1	1
Non-Saudi	0.98 (0.68–1.41)	0.82 (0.56–1.20)	0.75 (0.48–1.16)
**Gender**			
Male	1	1	1
Female	1.03 (0.78–1.37)	1.0 (0.75–1.34)	0.93 (0.68–1.28)
**Age (years)**			
15–25	1	1	1
26–35	1.70 (1.18–2.46) **	0.74 (0.51–1.06)	1.20 (0.79–1.80)
36–45	1.06 (0.71–1.60)	0.46 (0.29–0.69) **	0.97 (0.62–1.52)
>45	0.41 (0.26–0.64) **	0.22 (0.13–0.34) **	0.38 (0.23–0.63) **
**Education**			
High School	1	1	1
Bachelor	1.12 (0.65–1.93)	1.71 (0.93–3.13)	0.97 (0.54–1.74)
Master	1.58 (0.87–2.87)	2.07 (1.07–4.02) *	1.02 (0.53–1.97)
PhD	0.72 (0.38–1.35)	0.93 (0.46–1.88)	0.79 (0.39–1.56)
**Employment Status**			
Employed	1	1	1
Unemployed	1.04 (0.67–1.60)	0.99 (0.62–1.60)	1.0 (0.61–1.64)
Student	0.89 (0.64–1.25)	2.05 (1.48–2.84) **	0.99 (0.68–1.44)
Own Business	0.43 (0.16–1.12)	0.34 (0.10–1.14)	0.25 (0.06–1.05)
**Family Monthly Income**			
Low (<5000)	1	1	1
Average (5000–7000)	1.58 (0.99–5.54) *	0.78 (0.48–1.27)	1.72 (1.03–2.87) *
Moderate (8000–16,000)	1.22 (0.85–1.75)	0.61 (0.42–0.87) **	1.12 (0.75–1.68)
High (>16,000)	0.71 (0.48–1.04)	0.39 (0.26–0.58) **	0.71 (0.46–1.09)
**Supplement used during the quarantine**			
**Multivitamin**			
No	1	1	1
Yes	1.07 (0.77–1.49)	1.45 (1.04–2.02) *	1.45 (1.01–2.07) *
**Vitamin D**			
No	1	1	1
Yes	1.71 (1.22–2.39) **	1.53 (1.08–2.16) *	1.48 (1.02–2.15) *
**Selenium**			
No	1	1	1
Yes	0.01 (0.001–0.03)	0.01 (0.001–0.03)	0.02 (0.001–0.03)
**Vitamin C**			
No	1	1	1
Yes	1.49 (0.98–2.25)	1.29 (0.84–1.98)	1.63 (1.05–2.56) *
**Zinc**			
No	1	1	1
Yes	2.11 (0.56–7.91)	1.48 (0.37–5.95)	3.32 (0.88–12.5)
**Exposure to the sun during the quarantine**			
Yes	1	1	1
No	2.14 (1.43–3.21) **	2.10 (1.38–3.19)	1.75 (1.11–2.76)
Sometimes	1.16 (0.73–1.85)	1.34 (0.83–2.15)	1.39 (0.94–2.33)
**Sleeping hours increased during the quarantine**			
Yes	1	1	1
No	0.35 (0.25–0.48) **	0.26 (0.19–0.36) **	0.49 (0.35–0.69) **

Note: Data presented Odd Ratio (95% CI). * & ** represented *p*-Value < 0.05 and 0.01 level significant.

## Data Availability

The data used in this study is available on reasonable request from the corresponding author.
